# Our New York Letter

**Published:** 1890-11

**Authors:** Wm. L. Russell

**Affiliations:** New York


### Correspondence.

### OUR NEW YORK LETTER.

New York, October 15, 1890.

The subject of pneumonia in children was considered at the last meeting of the Section of the Academy on Pædiatrics. Discussion was especially invited on the following points: Is croupous pneumonia frequent in infancy? Does alcohol help or hinder recovery? Indications for antipyretics in pneumonia. Are there abortive cases?

The paper of the evening was read by Dr. Wm. L. Stowell, and consisted of an analysis of a hundred cases treated by him as visiting physician to Demilt Dispensary. The discussion was opened by Prof. Delafield, of the College of Physcians and Surgeons. He stated that in answering the first question upon which discussion had been invited, it was necessary to have a mutual understanding as to what was meant by the terms croupous pneumonia and broncho-pneumonia. In his mind, the difference was well marked, not in the absence or presence of bronchitis, for every pneumonia was accompanied by more or less bronchitis; not in the fact that there was consolidation of a whole lobe or only portions of a lobe, for the former occurred often in broncho-pneumonia, and the latter occasionally, although very rarely, in croupous pneumonia. The difference consisted in the character of the inflammation. In croupous pneumonia this was exudative in character, the blood vessels being almost solely concerned, it pursued a definite course of a limited period, and generally ended in recovery. In broncho-pneumonia, on the other hand, the inflammation was productive, resulting in the formation of new tissue cells around and within the bronchi and air-cells; the disease was likely to be protracted and to become sub-acute or chronic. This was the form of pneumonia of most frequent occurrence in children, but the croupous variety was also seen in a considerable proportion of cases. In adult life the reverse was true. In the intermediate period, or period of adolescence, both varieties were found in much more equal proportion.

In reply to the second question he would say that he had strong objections to the use of alcohol for children under five years of age under any circumstances whatever, and he believed that in pneumonia it usually did harm. To promote the comfort of the patient and allay nervous symptoms, he regarded antipyretic drugs as extremely useful, but for any other purpose the dose necessary was so large as to do harm.

Such cases as presented the history, and perhaps some of the physical signs, of croupous pneumonia and recovered in about two days, he regarded as examples of an exudative inflammation running a short course. The apparent abortion was probably not at all due to treatment.

Dr. J. E. Winters said, that accepting the definition of croupous pneumonia given by Prof. Delafield, the disease was comparatively frequent in children under two years old. In private practice among the better classes, broncho-pneumonia was rather infrequent. In dispensary and tenement-house practice, how ever, it wasvery common. He agreed with Prof. Delafield that alcohol usually did harm, and also with his views concerning the use of antipyretics. The question of abortive cases was exceedingly difficult to decide, owing to the impossibility of deciding from the symptoms just what the pathology of the condition was. He believed in the use of opium when there was much insomnia and restlessness, and spoke of it as advisable, useful and safe.

Prof. J. Lewis Smith believed that croupous pneumonia was very rare in children under three years. Broncho-pneumonia could often be aborted by the judicious use of emetics. He was not opposed to the use of alcohol in this disease, and had seen it produce good results when not given in too large doses. Two drops for every month of the child's age, every three or four hours, heregarded as a sufficient dose. It must be given on the same principle as other medicines, bearing in mind that an overdose would produce stupor and other ill effects. He was accustomed to use carbonate of ammonia, rather than the muriate. It was true that the former was apt to be irritating to the stomach, and might cause gastritis, but by giving it in milk this tendency was overcome. Frequent coughing was very important in order to free the air passages of muco-pus, and hence he believed opiates to be contra-indicated. Bromides were better to allay nervous irritability. It was important, too, to change the child's position frequently in order to prevent hypostasis.

The proper treatment of of peritonitis is a subject which has been considerably agitated here during the past year or more. Shall the opium treatment of Alonzo Clark give place to saline cathartics and operative interference in special cases. This question is by no means settled, as was demonstrated during a discussion on the subject at the last meeting of the County Medical Society.

Dr. H. C. Hanks, of the Woman's Hospital, read a paper. He stated that idiopathic peritonitis was rare, only three or four of his own cases seeming to have been such. Before deciding on this point the diagnostician must interrogate all the abdominal organs. The uterus, intestines, stomach, liver, spleen, and kidney should all be investigated in turn, careful percussion and palpation being employed. Rectal and vaginal touch should be added, sometimes with the assistance of an anæsthetic, which often aided materially in clearing up the case. The treatment which he generally adopted consisted in giving enough anodynes to relieve pain; and calomel a tenth of a grain tablet triturate every half hour, followed by a saline. Codeia was his favorite anodyne, as it did not constipate. The food should be liquid in character. Locally, an ice-bag placed on the abdomen, and even leeches or blisters, were useful in sthenic cases. If the temperature and pulse did not run higher under this plan of treatment he would persist; if the opposite were the case, however, or if other symptoms were increasing, laparotomy should be performed. Delay was fatal. If there were indications of pus the operation was always justifiable. It should be done by a special surgeon when such could be obtained.

Professor Jacobs being requested to give his views on the subject, as it related to children, stated that peritonitis was very frequent in infancy. It was often overlooked, many belly-aches being due to acute or sub-acute peritonitis or to acute exacerbations of the chronic variety. The last was probably the most frequent. This was proved by autopsies. In cases of perityphlitis for example, old adhesions were always found, and a rigid and patulous appendix was thus predisposed to allow the entrance of subtances from the bowels. Children often had numerous attacks. Where chronic peritonitis was known, or suspected to exist, a strong bandage should be worn at all times to steady the bowels. In acute cases he regared the opium treatment as superior to that by laxatives. Before the introduction of the Clark method, purges were always given, and now they were being re-introduced. They would certainly do harm if they did not benefit. Adhesions form early in these cases, and soon contain new vessels. Purges were liable to break these, and hemorrhages would result. This plan of treatment should then be confined to the very early stages. In using opium he believed in adhering to Clark's direction to keep the patient constantly asleep for the whole twenty-four hours.

Dr. W. H. Thomson drew attention to the fact that a great many cases of chronic Bright's disease ended with an attack of purulent peritonitis. In these cases there was often no rise of temperature. When peritonitis began with a sudden abdominal pain, the cause was usually perforative; when the pain advanced gradually from a small beginning, fæcal impaction or typhlitis was usually the cause.

Dr. C. C. Lee, of the Woman's Hospital, stated that as a gynecologist, he probably took a limited view of the subject under discussion. He thought, however, that in the case of septic peritonitis all were agreed concerning the advisability of laparotomy. He had been struck in a number of these cases by the singular absence of pain. The symptoms often simulated certain malarial conditions.

In acute cases, with obstruction of the intestinal canal, he had seen saline cathartics do good. If the cause was traumatism, a saline in the beginning, followed by enough codeia to quiet pain and peristalsis, was good treatment. Cold was admissible only during the formative stage, in the first twenty-four or forty-eight hours. When plastic exudation had occurred, cold did harm. Secondary peritonitis could only be affected by removal of the cause. The difficulty was to tell when not to operate, and he believed that operations would be done less frequently in the future than at present. WM. L. RUSSELL.

